# Hydrothermal Dehydration of Monosaccharides Promoted by Seawater: Fundamentals on the Catalytic Role of Inorganic Salts

**DOI:** 10.3389/fchem.2019.00132

**Published:** 2019-03-22

**Authors:** Maroua Kammoun, Thibaut Istasse, Haitham Ayeb, Neila Rassaa, Taoufik Bettaieb, Aurore Richel

**Affiliations:** ^1^Laboratory of Biomass and Green Technologies, University of Liege Gembloux Agro Bio-Tech, Gembloux, Belgium; ^2^Louvain Institute of Biomolecular Science and Technology, University of Louvain, Louvain-la-Neuve, Belgium; ^3^Laboratory of Agricultural Production Systems Sustainability in Northern Region of Tunisia, University of Jendouba, Le kef, Tunisia; ^4^Laboratory of Horticultural Sciences, University of Carthage National Agronomic Institute of Tunis, Tunis, Tunisia

**Keywords:** D-glucose, D-xylose, dehydration, seawater, inorganic salts, hydrothermal, lactic acid, levulinic acid

## Abstract

In biorefining, the conversion of carbohydrates under subcritical water conditions is a field of extensive studies. In particular, the hydrothermal decomposition of benchmark C6- and C5-monosaccharides, i.e., D-glucose and D-xylose, into furanics and/or organic acids is fully considered. Herein, we propose to establish the fundamentals of the decomposition of D-glucose and D-xylose under subcritical water conditions in the presence of specific salts (i.e., NaCl and KI) and in seawater. Our results demonstrated that the introduction of inorganic salts was found to modify sugars dehydration yields. Different NaCl concentrations from 0.21 to 1.63 mol L^−1^ promoted the conversion of D-xylose to 2-furfural (2-F) from 28 to 44% (molar yield). NaCl also improved 5-hydroxymethylfurfural (5-HMF) generation from D-glucose as well as rehydration of 5-HMF to levulinic and formic acid. KI favored other pathways toward formic acid production from D-glucose, reaching 20% in the upper concentration. Compared to a solution of equivalent NaCl concentration, seawater enhanced selectivity toward lactic acid which was raised by 10% for both monosaccharides, and sugars conversion, especially for D-glucose whose conversion was increased by 20%. 5-HMF molar yield around 30% were achieved from D-glucose in seawater at 211°C and 20 bars after 15 min.

## Introduction

Upgrading of biomass is a field of extensive efforts. In particular, its hydrothermal conversion into valuable products has been extensively proposed in the state of the art (Tekin et al., 2014).

In fact, Biomass represents a renewable resource contributes to the development of a new-bioeconomy and to sustainable chemical industry. Several literatures reported the production of biobased molecules, from various sources such as giant reed (Licursi et al., 2018b), corncobs (Peleteiro et al., 2018), eucalyptus globulus wood (Peleteiro et al., 2018; Rivas et al., 2018), hazelnut shells (Licursi et al., 2017), and fructose (Antonetti et al., 2017). In these studies, the conversion carried in hydrothermal media using acid hydrolysis mostly with hydrochloric, sulfuric acids and acid resin (Amberlyst-7) in a microwave and autoclave reactors. The synthetic route employs monosaccharides as starting substrate, which, under hydrothermal conditions, undergoes dehydration to form the biobased molecules.

The understanding of the fundamentals of the conversion of “model” monosaccharides (including representative C5 sugars as D-xylose or C6 entities as D-glucose, D-fructose or D-mannose) under subcritical water conditions (namely between 100 and 374°C at a pressure high enough to maintain the liquid state) is still under investigations as the mechanisms governing the monosaccharides decomposition are complex and numerous (Srokol et al., 2004).

**Figure 1:**
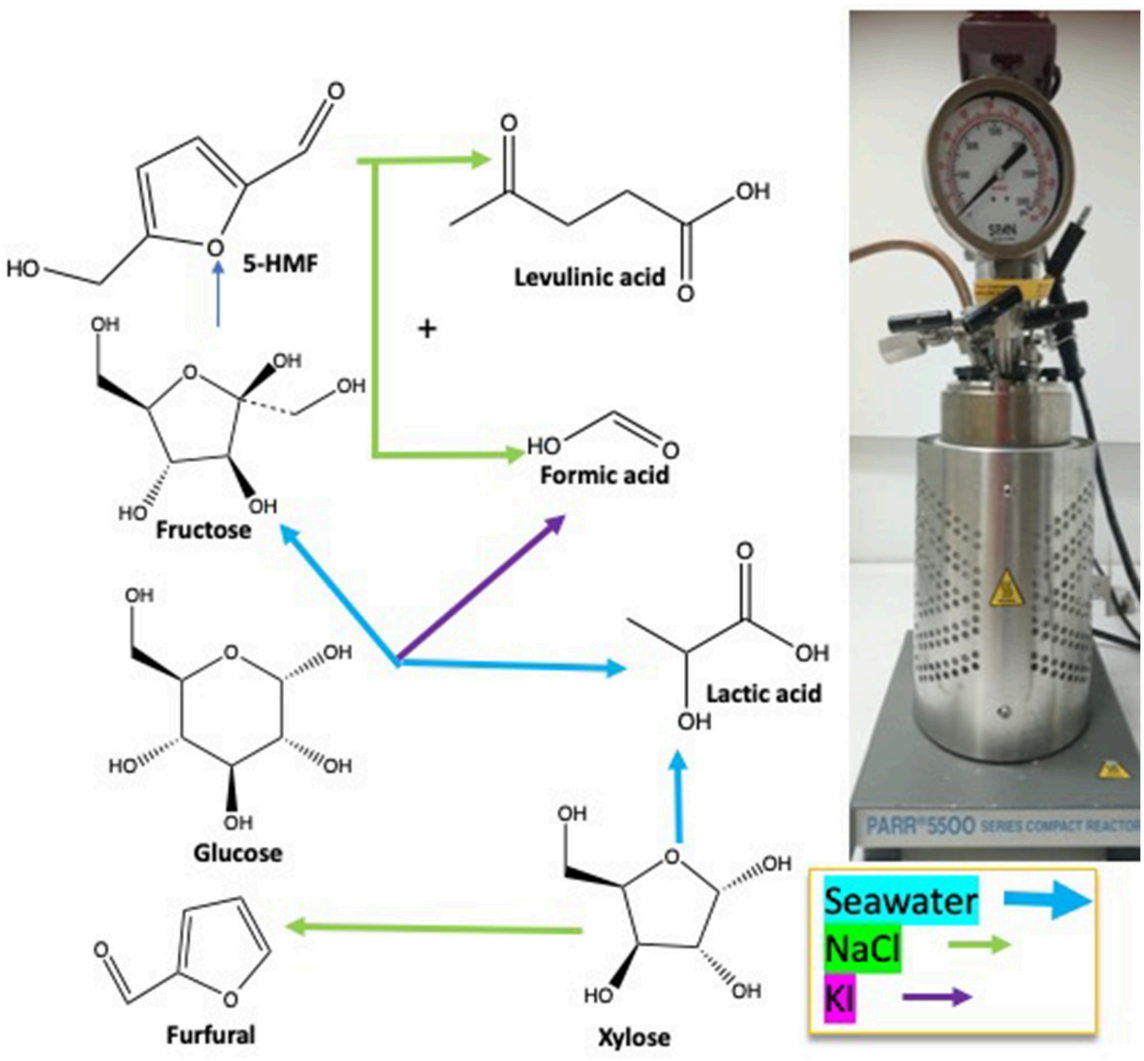
D-xylose conversion pathways in subcritical water adapted from the works of Aida et al. (2010), You et al. (2014), and Delbecq et al. (2018). Reactions: D, dehydration; P, polymerization; BR, benzilic acid rearrangement; I, isomerization; RA, retro-aldol.

**Figure 2:**
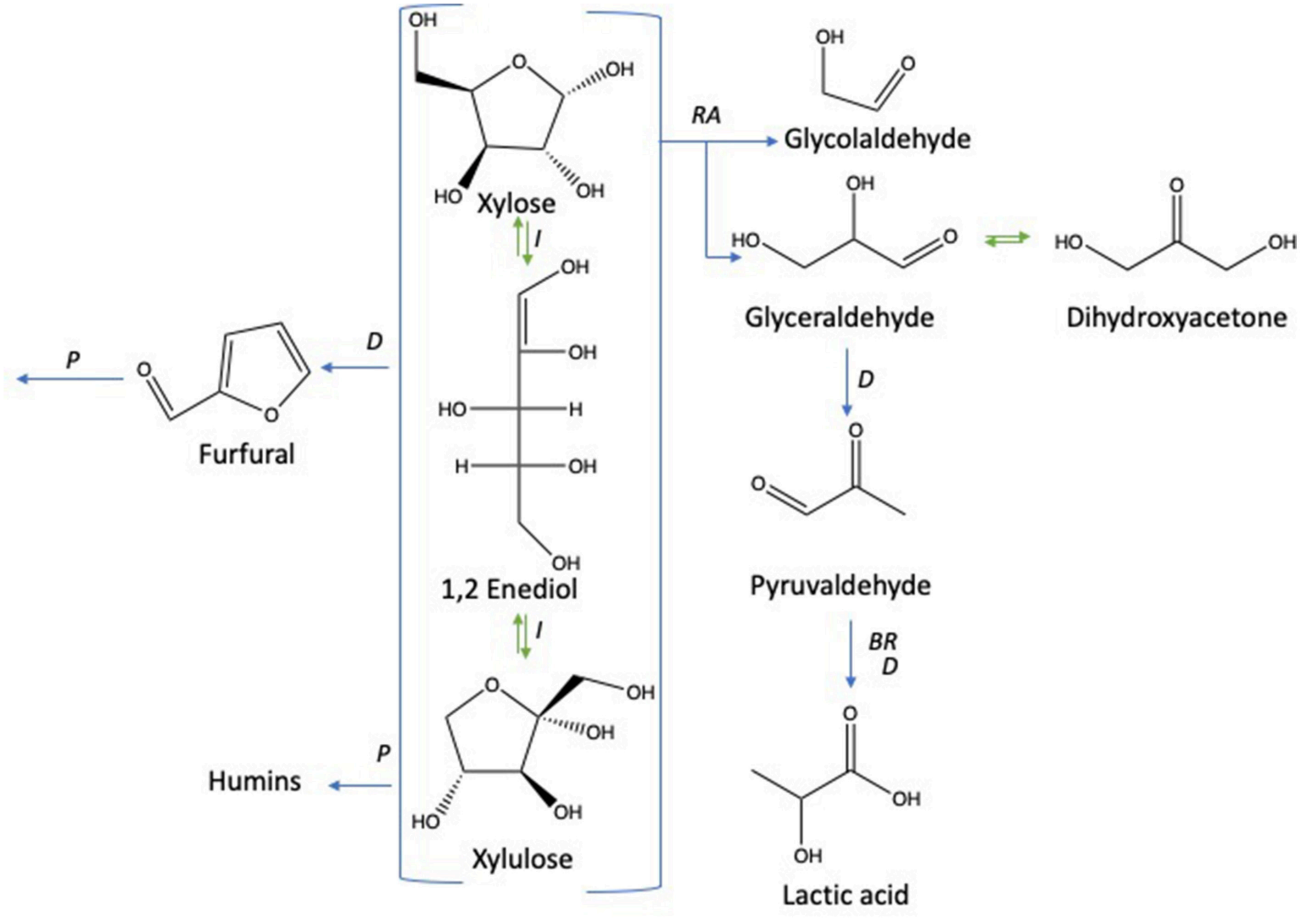
D-glucose conversion pathways in subcritical water adapted from the works of Kabyemela et al. (1999); Watanabe et al. (2005); Rasrendra et al. (2010); Assary et al. (2012), and Aida et al. (2007). Reactions: D, dehydration; P, polymerization; BR, *benzilic acid* rearrangement; I, isomerization by Lobry de Bruyn–van Ekenstein transformation, RA: retro -aldol, H: hydration.

Under subcritical water conditions, it is nowadays well-established that monosaccharides undergo numerous reactions like isomerization, condensation into disaccharides, dehydration, and fragmentation. The dehydration of C6- and C5-monosaccharides typically leads to the respective formation of 5-hydroxymethylfurfural (5-HMF) and 2-furfural (2-F) together with variable amount of organic acids (resulting partially from the subsequent rehydration of furanic compounds) together with variable amounts of humins (Kabyemela et al., 1999). Fragmentation reactions result in the formation of erythrose, glycolaldehyde, and glyceraldehyde, which yields low molecular weight organics including acetic acid and lactic acid as final products. pH conditions strongly impact monosaccharides conversion pathways. Dehydration paths are favored in acidic conditions while the formation of glycolaldehyde and glyceraldehyde as well as isomerization are mainly base-catalyzed mechanisms. The major conversion pathways of D-xylose and D-glucose in subcritical water are depicted in Figures 1, 2, respectively.

Among the products presented in Figures 1, 2, furanic compounds and levulinic acid are the most promising to develop new bio-derivatives (i.e., solvents, fuels, and polymers) (Bozell and Petersen, 2010). Levulinic acid is easily produced through different processes such as biphasic [paraffin oil/water] (Licursi et al., 2018c), acid catalyst (Rivas et al., 2015, 2016) catalytic system [Ru/C + zeolite HY] (Licursi et al., 2018a). These studies investigated the important potential to convert biomass to value-added product, in particular the biobased platform molecules and their promising applications.

With the ambition to accelerate monosaccharides conversion under hydrothermal conditions and/or to improve the selectivity of this conversion, some studies have therefore reported the introduction of Brönsted acid catalysts (typically HCl or H_2_SO_4_) or bases (sodium hydroxide notably), and/or specific inorganics salts (Assary et al., 2012; Li et al., 2017). Indeed, it has been described that specific ions (i.e., metal cations as Al^3+^, Cr^3+^, Zn^2+^, Cu^2+^, Ag^+^, etc.) enhance the conversion rate of C6-monosaccharides into 5-HMF (Wang et al., 2015). In particular, MgCl_2_ was found to increase significantly the formation of levulinic acid when using HCl as the acid catalyst. Zn^2+^ or Co^2+^ salts were found to increase the formation of lactic acid. Due to the ability of Lewis acid to convert triose like glyceraldehyde and dihydroxyacetone to lactic acid (Rasrendra et al., 2010). The ionic strength of the reaction medium was also reported as a key point for modulating the selectivity and kinetics of the dehydration of D-glucose into 5-HMF. In this sense, (Tang et al., 2017) reported on the impact of the adjunction of sodium chloride to a biphasic THF/water system. The formation of hydrogen bond between the ion Cl^−^ and glucose in the position (C6)O-H leads to a ring-opening of glucose and to a mutarotation from α-D-Glucose to β-D-Glucose. Nevertheless, in the NaCl reaction media, the ratio of β-D-glucose/α-D-glucose at mutarotation equilibrium is lower than that without NaCl. This affect glucose by decelerating its isomeration to fructose and suppressing its polymerization of humins. Actually, extensive R&D efforts are directed on the evaluation of biphasic or triphasic (organic) media as well as the selection of options as ionic liquids, deep eutectic solvents, etc. However, it appears that water plays a crucial role in the monosaccharide dehydration, catalyzing proton transfer and speeding up so the reaction (Zhou et al., 2017). Besides their influence on monosaccharides conversion pathways, inorganic salts also improve partition coefficients during the extraction of 5-HMF, 2-F and levulinic acid, which is of primary importance for purification steps (Sindermann et al., 2016).

As a cheap, abundant and salts rich medium, seawater could present interesting qualities for monosaccharides conversion. This natural source of salts ions (i.e., Na^+^, Cl^−^, K^+^, Mg^2+^) could be used as a processing medium at large scale especially for coastal cities while preserving drinkable water resources. Seawater is nowadays no longer limited to salts and seafood production. Due to its great potential, biorefinery concepts could benefit of it for aqueous-based processes as suggested in previous works. Lin et al. (2012) proved that seawater is a suitable medium for *Actinobacillus succinogenes* growth and also offers a complete nutrient medium for succinic acid production. Some papers investigate seawater as a solvent in furfural production processes using different catalysts, such as FeCl_3_, NaCl, oxalic, and acetic acids, in which furfural production rate was enhanced (Vomstein et al., 2011; Grande et al., 2012; Mao et al., 2013; Hongsiri et al., 2014). Moreover, seawater would supply the appropriate source of sodium chloride for effective sugar dehydration. Chloride ions promote the formation of 1,2-enediol from the acyclic form of D-xylose, which is then dehydrated to furfural in the presence of acid catalysts (Marcotullio and De Jong, 2010).

KCl, CaCl_2_ and MgCl_2_ have also been used as main catalysts or co-catalysts for efficient sugar dehydration (Liu and Wyman, 2006; Zhou et al., 2014). However, the effect of potassium iodide on monosaccharides dehydration has not been studied yet in deionized water. Also, in biorefinery, hydrothermal treatment of some biomasses, such as halophyte plants in pilot reactor like steam explosion, allows the extraction of some components among which salts and monosaccharides. Some interactions are established between extracted sugars and salts during pretreatments and form other products like lactic acid, formic acid, levulinic acid, 2-furfural, 5-HMF. Depending on the envisaged application, their generation is favored or disadvantaged. In bioethanol production, these resulting products are avoided since they are considered as inhibitory compounds for microorganism during enzymatic hydrolysis. Contrariwise they are considered as platform products for chemical production.

In this study, pentose and hexose dehydration to furanic compounds and organic acids were investigated in the presence of inorganic salts in hydrothermal conditions at 211°C. The main salts found in biomass and seawater, i.e., NaCl and KI, were selected as benchmarks for this fundamental study. The objective is to assess the decomposition features of D-glucose and D-xylose in different mixtures of salts: (i) both aqueous solutions of sodium chloride and potassium iodide at increasing salts molarity from 0.21 to 1.63 mol L^−1^; (ii) seawater from different geographical origins without additional inorganic catalysts.

## Experimental Section

### Materials

D-glucose (98%), D-xylose (98%), and D-fructose (99%) were supplied by Sigma Aldrich lactic acid (88%) from VWR, formic acid (99%) from Biosolv LTD, levulinic acid (98%), 5-hydroxymethylfurfural (5-HMF 98%) and 2-furfural from Alfa Aesar (98%), NaCl and KI were purchased from VWR and used as received.

**Figure 3:**
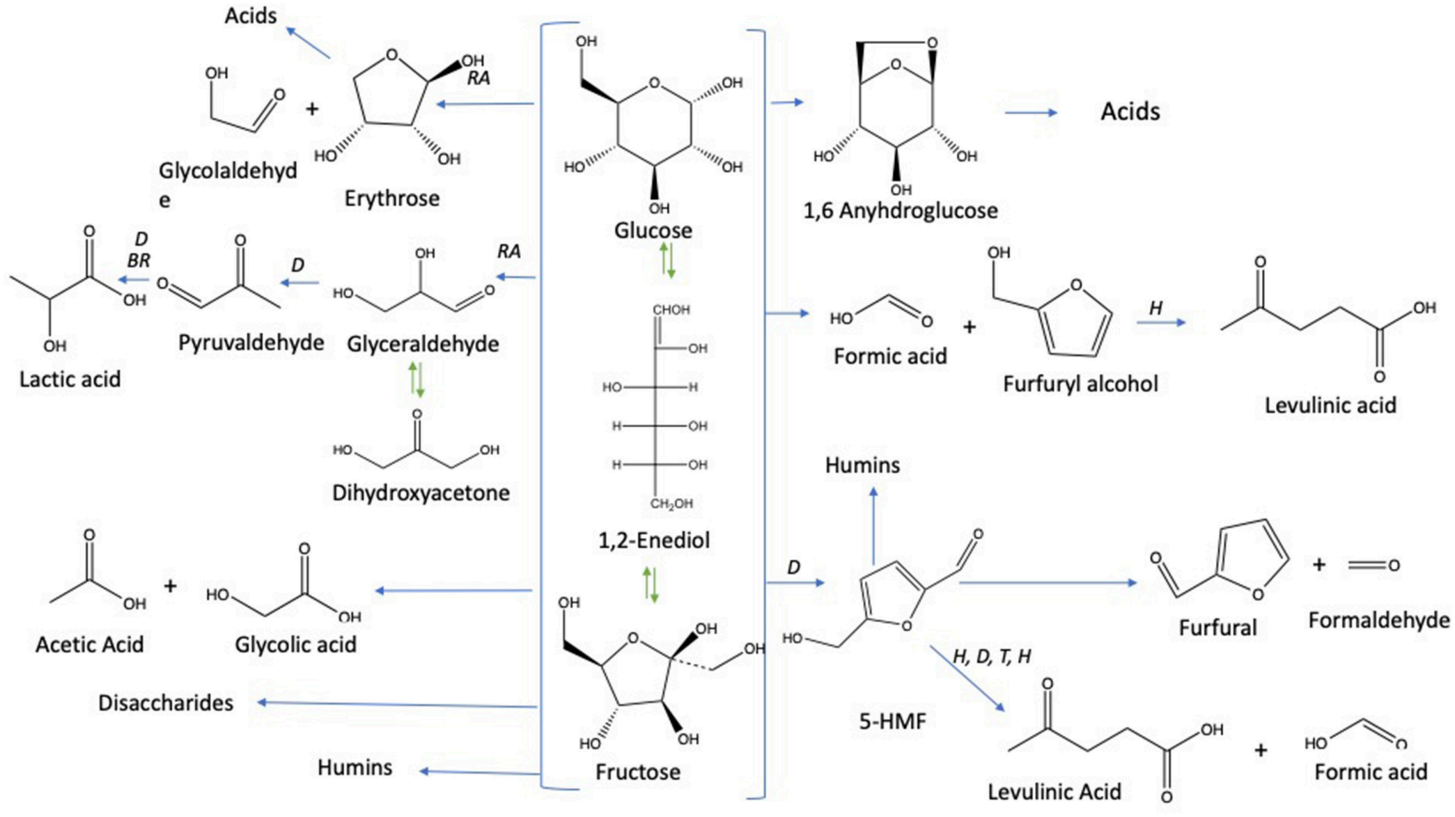
Seawater samples locations. **(A)** Zeland, **(B)** Kerkenah, **(C)** Salakta.

Seawater samples were collected from three different locations presented in Figure 3 between October and December 2017: Zeeland in the Netherlands (pH 8.04), Salakta in Tunisia collected (pH 7.93) and Kerkenah in Tunisia too (pH 8.19). Seawater samples characterization was carried out in a Laboratory called “Green lab” in Tunisia which is accredited by TUNAC according to ISO/IEC17025 standard. The composition of seawater samples is reported in Table 1.

**Table 1:** Seawater samples composition by Green lab-TUNAC-Tunisia (All parameters are expressed in mg L^−1^).

**Seawater**	**Dry Residues**	**Chlorides**	**Sodium**	**Sulfates**	**Magnesium**	**Potassium**	**Calcium**
Zeland	38.4 10^3^	18.3 10^3^	11.3 10^3^	2.66 10^3^	1.48 10^3^	359	425
Kerkanah	43.2 10^3^	21.0 10^3^	13.3 10^3^	2.41 10^3^	1.61 10^3^	398	419
Salakta	36.4 10^3^	18.2 10^3^	11.1 10^3^	2.26 10^3^	1.39 10^3^	339	370

### Monosaccharides Conversion to Organic Acids and Furanic Compounds

The hydrothermal decomposition of monosaccharides (D-xylose and D-glucose) was performed in a Series 5,500 Parr reactor (600 mL) which is equipped with a temperature probe connected to a 4,848 Parr reactor controller and a mechanical stirring. The reactor was heated during 40 min to reach a target temperature of 211°C which was then maintained during 15 min. At the end of the treatment, the reactor was cooled down until 40°C (Figure S1). This process has been realized for monosaccharides (70 g/L) in deionized water, in solutions of NaCl or KI (from 35 to 70 g/L), in solutions containing a mixture of NaCl and KI and also in seawater. Each experiment was performed in triplicate. Before analysis, samples were filtered with 0.45 μm syringe filter and diluted with distilled water.

### Analytic of Methods

Monosaccharides (D-xylose, D-glucose, and D-fructose) were determined using a high-performance liquid chromatography (HPLC) system Dionex 5,000 with acarbo Pac PA-100 (4 × 250 mm) anion exchange column and pulsed amperometric detection. Four different eluents were used: (A) 100 mM NaOH, (B) 100 mM NaOH + 600 mM NaCl, (C) 500 mM NaCl, and (D) ultra-deionized water. The decomposition products were analyzed using high performance liquid chromatography coupled with UV detection at 210 nm for organic acids and 284 nm for furanic compounds. 5 mM sulfuric acid was used as the elution solvent with a flow rate of 0.6 mL/min on a Aminex HPX-87H column (300 × 7.8 mm) at 45°C (Istasse et al., 2018).

The yield of all the compounds were defined by calculating the molar ratio between the generated product and the initial monosaccharides concentration (Figure S2).

### Chemical Structure

Molecules were drawn using Chem Draw professional 15 (http://www.perkinelmer.com/fr/product/chemdraw-professional-chemdrawpro).

### Data Treatment and Statistical Analyses

Results presented in Violin plot figures are the means of nine replicates. Scatter plot and Anova one-way analysis of variance as well as the Bartlett's test for equal variances were achieved using Graphpad Prism 7 (https://www.graphpad.com/scientific-software/prism/).Means were compared according to Tukey's multiple comparison test. The principal component analysis (PCA) was done using XLSTAT software (https://www.xlstat.com/fr/). Correlation matrix were realized using R Corrplot package. The geographic map was developed by integrating spatial data of sea zone map using ArcGIS (ArcInfo edition, ESRI, Redlands, CA: https://www.arcgis.com/index.html).

## Results and Discussions

### Hydrothermal Conversion of Monosaccharides in Deionized Water

**Figure 4:**
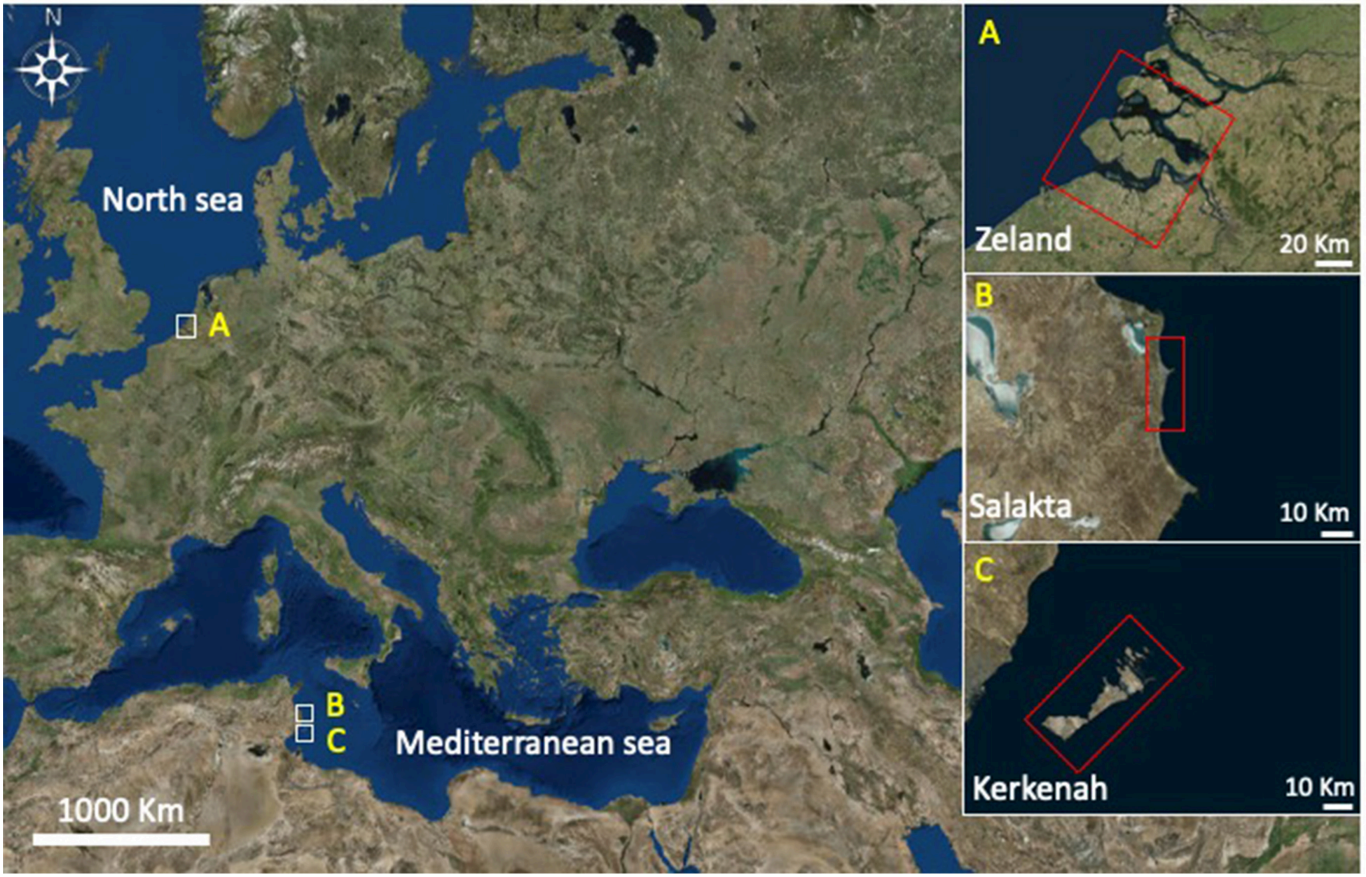
Hydrothermal decomposition of D-glucose to formic acid and furfuryl alcohol (Assary et al., 2012). Reactions: Pr, protonation; H: hydration; D, dehydration; DPr, deprotonation.

The dehydration of D-glucose and D-xylose in deionized water was selected as the benchmark and was achieved in a high-pressure reactor at 211°C corresponding to an internal reactor pressure of 20 bars. A preliminary kinetic investigation allowed to identify that 15 min was the optimal reaction time required to obtain the maximum yields in 2-furfural (2-F) and 5-hydroxymethylfurfural (5-HMF) in deionized water from respectively D-xylose and D-glucose (Figure S3). Classically, it appeared that D-glucose underwent a dehydration path with a successive formation of D-fructose through isomerization and subsequent dehydration into 5-HMF, whilst D-xylose was dehydrated in a single step into 2-F′. Figures S4 and S5 show the HPLC_UV chromatograms for products obtained from the reaction of D-glucose and D-xylose, respectively, in deionized water at 211°C. An average molar yield of 19.61% was recorded for 5-HMF from D-glucose compared to about 28.05% for 2-F from D-xylose. Rehydration of these molecules was expected to generate a mixture of both levulinic (LA) and formic acids (FA). At first sight, it appeared however that LA was not detected in the reaction media, whilst FA accounted for more than 11% when investigating D-glucose (FA was not detected in the case of D-xylose). This observation supports that other major pathways are responsible for formic acid generation in deionized water as mentioned in Figure 2. (Assary et al., 2012) proposed a mechanism initiated by the protonation of the glucose at the (C_2_)-OH group which leads to the production of furfuryl alcohol and formic acid. A series of reactions take place including a dehydration followed by the hydration to the aldehyde group. Then, a deprotonation gives a gem-diol which undergoes a C-C scission to release the formic acid and a thermodynamically stable intermediate (5-hydroxymethyl)tetrahydrofuran-2,4-diol. This reaction pathway leads to the production of furfuryl alcohol (Figure 4). This latest compound can also undergo rehydration into levulinic acid. The absence of 5-HMF rehydration in the tested conditions suggests however that furfuryl alcohol could be stable enough, which could explain the formation of formic acid without levulinic acid. The pH of the solution shifted from 6 to 3 after the treatment of D-xylose and D-glucose. Lactic acid was also detected as a by-product with a substantial production reaching 13.36 and 7.16% for D-glucose and D-xylose, respectively.

### The Effect of Inorganic Salts Concentration on D-xylose Conversion

**Figure 5:**
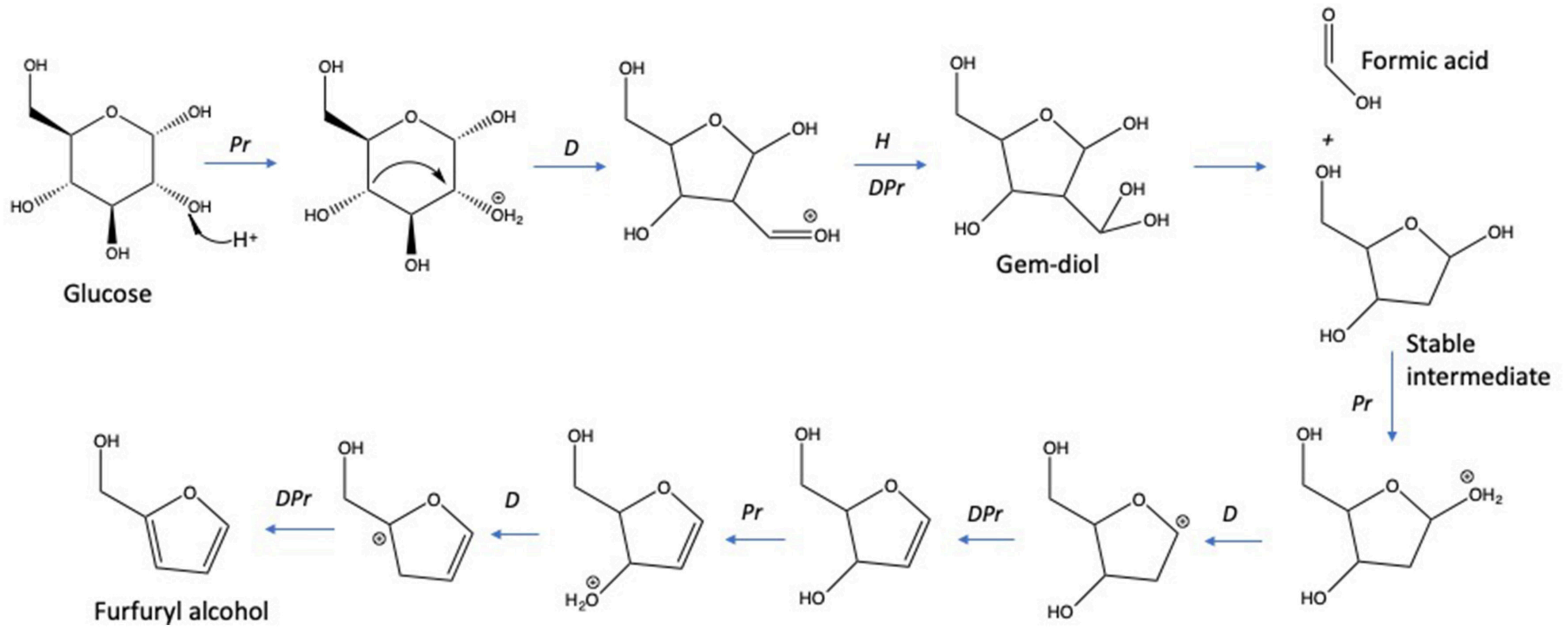
The effect of adding NaCl and KI in hydrothermal dehydration of D-xylose: **(A)** molar conversion efficiency of D-xylose **(B)** molar conversion efficiency into lactic acid and **(C)** molar conversion effeciency into furfural **(C)**. Molarities 0; 0.21; 0.42; 0.6; 0.81; 1.19; 1.63 corresponding g/L to deionized water 35KI; 70KI; 35NaCl; 35 KI+ 35NaCl; 70NaCl; 70KI+70NaCl, respectively. Significant differences were determinated with on-way ANOVA and Tukey HSD *post hoc* test (*P < 0.05; **P < 0.01; ***P < 0.001).

Experiments were conducted to probe the role of NaCl and KI in D-xylose dehydration into 2-furfural and lactic acid. Figures 5A–C depict xylose conversion, the lactic acid yield and the 2-F yield under the reaction temperature at 211°C for 15 min. D-xylose conversion significantly increased with the overall molar salt concentration shifting from 67% when dehydration was performed in deionized water to more than 90% when introducing salts. Conversion was also boosted when NaCl and KI molarity increased. Addition of sodium chloride at concentrations of 35 and 70 g/L improved the formation of 2-F from D-xylose reaching a yield of about 43% for the upper NaCl concentration. This is in accordance with the results of (Marcotullio and De Jong, 2010), which show that Cl^−^ ions promote 1,2-enediol formation and thus dehydration to furfural. Levulinic acid and formic acid were not detected. Surprisingly, KI showed a different effect on 2-F production, a higher KI concentration seemed to significantly decrease 2-F yield. Lactic acid was also monitored with a production ranging from 2 to 6%. Lactic acid yield was significantly reduced by increasing molar concentration for both salts, namely NaCl and KI.

### The Effect of Inorganic Salts Concentration on D-glucose Conversion

**Figure 6:**
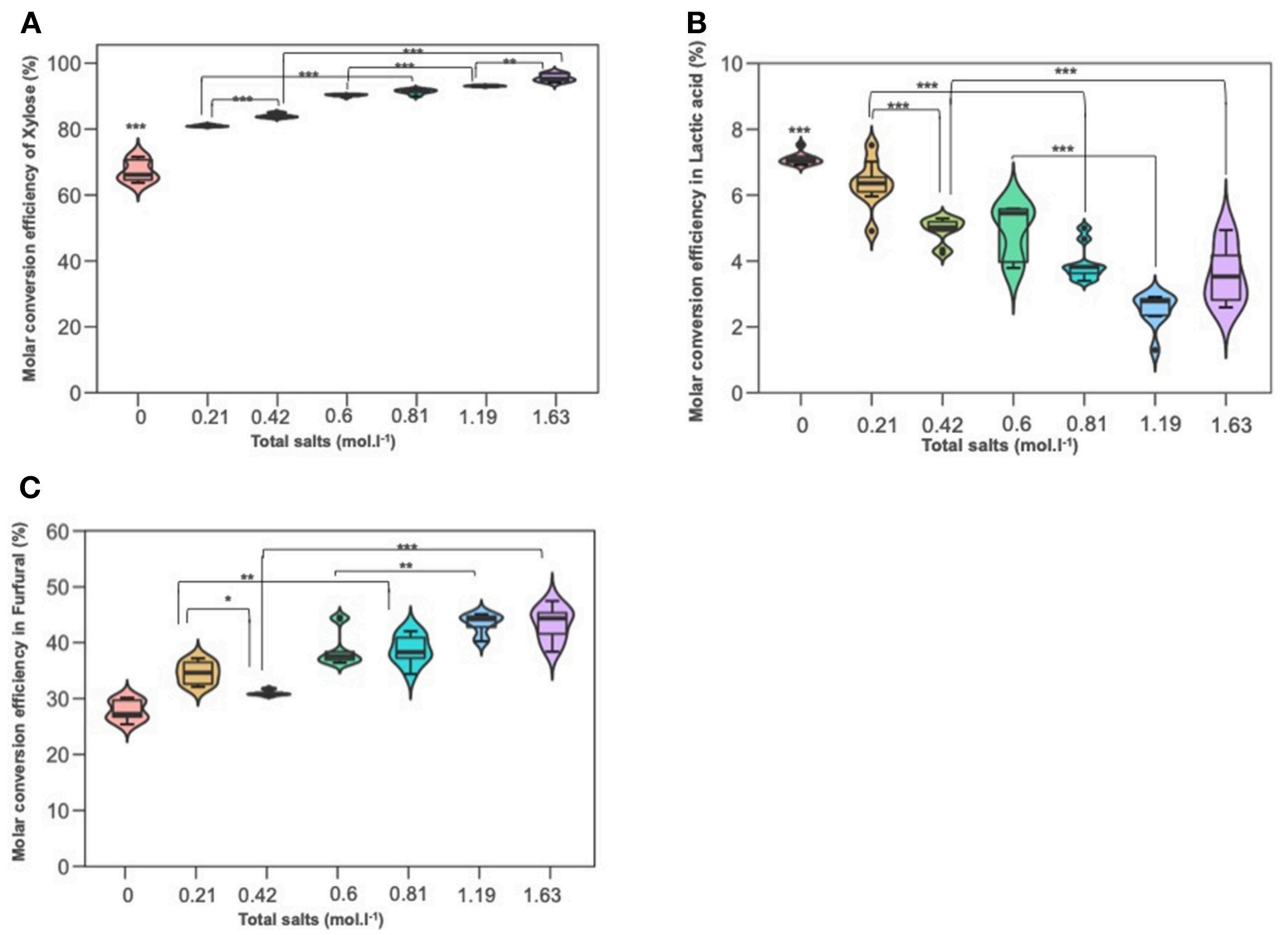
The effect of adding NaCl and KI in hydrothermal dehydration of D-glucose: **(A)** molar conversion efficiency of D-glucose, **(B)** molar conversion efficiency to fructose, **(C)** molar conversion efficiency to lactic acid, **(D)** molar conversion efficiency to Formic acid, **(E)** molar conversion efficiency to levulinic acid, and **(F)** molar conversion efficiency to HMF. Molarities 0; 0.21; 0.42; 0.6; 0.81; 1,19; 163 corresponding g/L to deionized water; 35KI; 70KI; 35NaCl; 35 KI+ 35NaCl; 70NaCl; Q19 70KI+70NaCl, respectively. *P < 0.05; **P < 0.01; ***P < 0.001.

When the hydrothermal treatment of D-glucose was investigated in the presence of the same salts, we found that conversion was also significantly higher compared to deionized water. The results are demonstrated in Figures 6A–F. D-glucose reactivity was enhanced at high NaCl molarity. Similar D-glucose conversions were achieved in KI at 0.21 mol L^−1^ and NaCl at 0.60 mol L^−1^ (75.39 and 74.88%, respectively). Concerning D-fructose, no significant effect was detected in salts treatment, under different concentrations, and deionized water. Its yield was low and did not exceed 0.7%. 5-HMF yield significantly increased in upper concentration 70 g/L of both KI and NaCl compared to their lower 35 g/L. Levulinic acid (LA) was not detected in KI processes, while it was detected in NaCl treatments and its yield was significantly higher in the upper concentration 70 g/L (1.19 mol L^−1^) than the lower 35 g/L (0.6 mol L^−1^). Lactic acid quantification through HPLC afforded values ranging from 7 to 11% as in terms of the inorganic salt used for the hydrothermal treatment. Variance analysis (ANOVA) showed that its yield was significantly lower than that in deionized water, while formic acid (FA) showed the opposite trend: significantly higher in both NaCl and KI processes whose yields were noticeable culminating at more than 15% and reaching up to 20% for the upper KI concentration. According to Figure 2, the rehydration of 5-HMF produces a fair ratio of LA and FA. If the majority of LA is obtained from 5-HMF rehydration and considering that no LA was produced in distillated water, the addition of salts improves the dehydration of D-glucose. By summing produced moles of 5-HMF and LA, it was found that the overall generated 5-HMF increased with salts concentration until 0.81 M where a plateau is reached. The total production of 5-HMF for this salt concentration can be estimated at around 27%. Since LA is observed in the presence of NaCl in contrast to distillated water and KI solution, it is likely that NaCl promoted 5-HMF rehydration. When performing reactions in both NaCl or KI media, the pH tended to decrease due to the formation of organic acid side-products, i.e., LA and FA. The acidity of the reaction media after the process was quite stable whatever the monosaccharide or the nature of the inorganic salt. Indeed, the pH value was measured at a constant value of about pH 2.5.

### Effect of Salts Combination on D-xylose Conversion

To better understand the different effects of the previous salts on monosaccharides degradation, a combination of KI and NaCl was done, first with the lower concentration 35 g/L and second with the upper concentration of 70 g/L of each salts.

According to Figures 5A–C, it can be seen that the conversion of D-xylose was significantly increased parallelly with the increased total molarity of salts. It was boosted by 10% and 12% in 0.81 mol L^−1^ (the lower combination solution) and at 1.63 mol L^−1^ (the upper combination solution) compared to KI solution at 0.21 and 0.42 mol L^−1^, respectively. Besides, the higher the salt concentration, the lower the lactic acid detection. The variance analysis showed that lactic acid yield in combination solutions (0.81 and 1.63 mol) was significantly lower than that in KI solutions and which is not the case for NaCl, this can be explained maybe by the effect of sodium chloride on the appearance of some modifying functional groups to the intermediate molecule glycolaldehyde and pyruvaldehyde. Furfural yield was improved with increased salt concentration. Compared to KI solutions (0.2 and 0.42 mol L^−1^), 2-F increased significantly in the mixture salt at 0.81 and 1.63 mol L^−1^, which is not the case in NaCl solution and so this highlights that NaCl has a higher effect than KI salt on D-xylose dehydration to furfural production.

### Effect of Salts Combination on D-glucose Conversion

Figures 6A–F show the effect of the amount of salts on D-glucose dehydration. Similarly to xylose conversion, the conversion of glucose increased parallelly by increasing the molarity of salts and reached 84%. Fructose yield remained low and no significant differences between treatments. As expected, lactic acid was significantly decreased by increasing salts molarity recording 3% for 1.63 mol L^−1^. These results revealed that salts concentration had an antagonistic effect on lactic acid production for pentoses as well as hexoses. LA which was not detected in KI processes, was identified in the presence of salts mixture. Variance analysis showed that its yield was significantly increased in upper combination (1.19 mol L^−1^) compared to the lower combination (0.81 mol L^−1^). This result was in agreement with those previous results of NaCl processes. Formic acid yield was up to 20% in the upper combination (1.63 mol L^−1^) which gave the same yield in KI process in upper concentration (0.42 mol L^−1^). This yield was significantly higher than that in NaCl processes. This result stuck very well with previous results of KI.

### Seawater's Effect on Xylose Conversion and Dehydration

**Figure 7:**
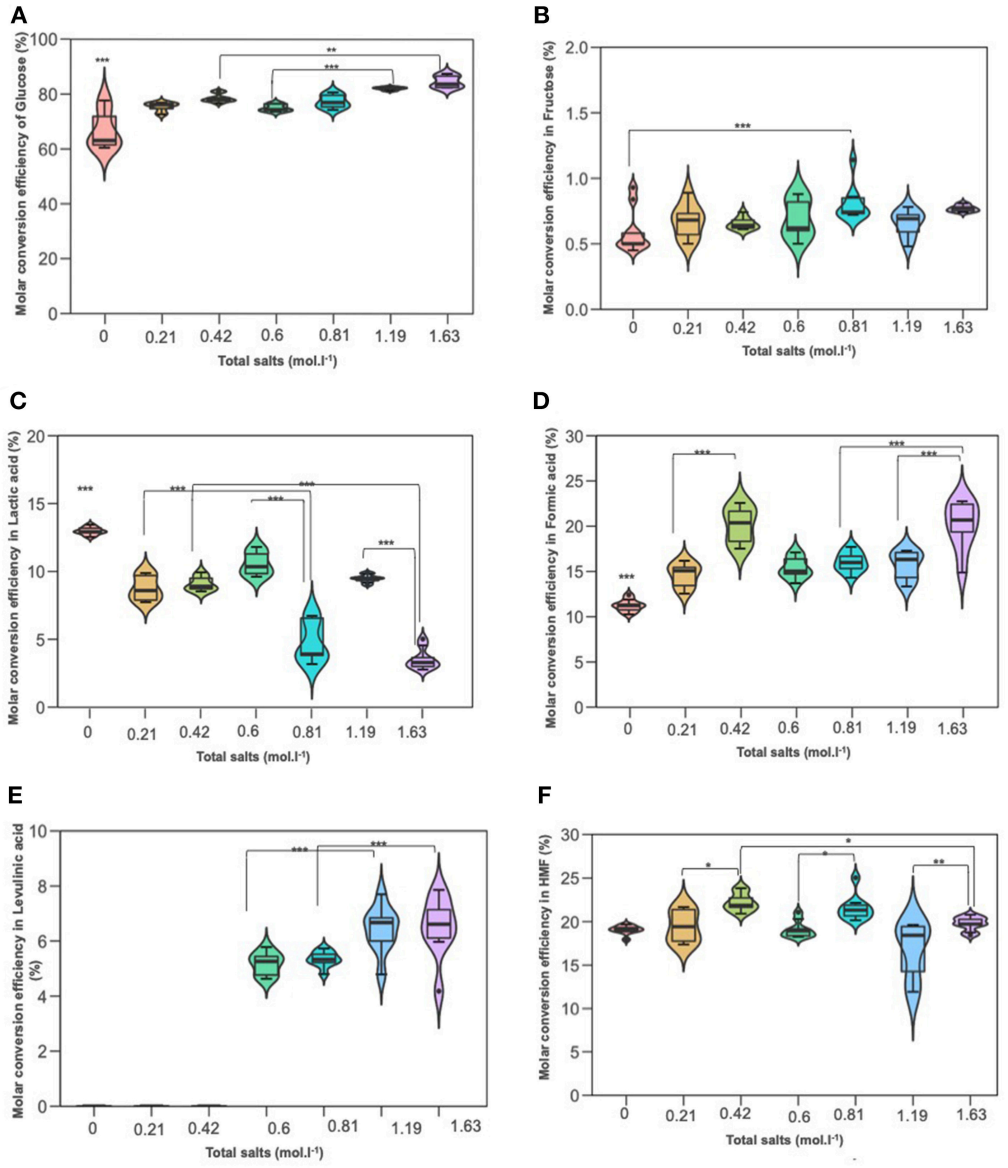
The effect of inorganic salts and seawater on D-xylose and D-glucose conversion.

Further experiments were performed under the same conditions at 211°C for 15 min in a natural source of salts, cheap and abundant, the seawater. Table 1 highlights samples compositions in main salts. Molarities, been estimated on the basis of chloride concentration (mass concentration of chlorides / molar mass of chlorine). Zeland and Salakta seawater had almost the same molarity 0.51 and 0.52 mol L^−1^, respectively, while Kerkenah was 0.59 mol L^−1^. Figure 7 recaps the effects of three seawaters samples on D-xylose dehydration compared to previous treatments. Interestingly, the yield of lactic acid was remarkably enhanced. Figures S6A–C show the effect of seawater samples on the D-xylose conversion, the 2-F and lactic acid yield. Conversion of D-xylose was higher than 96% for the three samples of seawater, which was significantly higher compared to distilled water. ANOVA showed that sugar conversion was also significantly higher not only compared to that in KI and NaCl solutions for concentrations < 0.5 mol L^−1^ (0.21 mol L^−1^; 0.42 mol L^−1^; 0.6 mol L^−1^) but surprisingly comparable to the results obtained in combined solution (0.8 mol L^−1^; 1.19 mol L^−1^). This observation suggests that other factors than salt concentration are responsible for sugar conversion in seawater. The yield of 2-F reached around 31% for Kerkenah which was significantly higher compared to Zeland, Salakta, and deionized water. This yield is consistent regarding the previous results achieved at different NaCl concentrations. Seawater also favored lactic acid production with yields reaching 14 to 17% for Zeland and Kerkenah, respectively. Variance analysis showed that these yields were significantly higher to lactic acid yield produced in deionized water and different salt concentrations. De Bruijn et al. (1986) indicated that calcium ions enhance lactic acid production by increasing monosaccharides retro-aldolization.

### Seawater's Effect on D-glucose Conversion and Dehydration

Figure 7 and Figures S7A–C represent the effect of seawaters samples on D-glucose conversion into furanic compounds and organic acids. Regarding D-glucose reactions in seawater, the conversion yield was significantly higher compared to deionized water and different salts concentrations. More than 94% of D-glucose was converted in seawater while only 81.98% of D-glucose was converted in the NaCl solution at 1.19 mol L^−1^. The highest yield of levulinic and formic acid were detected in Kerkenah seawater compared to Zeland and Salakta seawater, but it remained significantly lower than the yield recorded for NaCl solutions. Compared to distillated water and salts solutions, 5-HMF formation was significantly improved in seawater media. Again, with the assumption that levulinic acid is generated from 5-HMF rehydration, overall 5-HMF yields higher than 30% are estimated. Lactic acid yield was also significantly enhanced compared to deionized water and salts solutions, because of calcium ions as previously explained for D-xylose. The highest yield was recorded in Kerkenah seawater relative to Zeland and Salakta seawater. Moreover, D-fructose molar yields of ~4% were observed in seawater which was significantly higher related to previous treatment. D-fructose could imply that the isomerization rate is higher in this medium compared to deionized water or tested saline solutions. Several factors could promote isomerization including the higher initial pH of seawater which could favor the Lobry de Bruyn–van Ekenstein transformation (Kobayashi et al., 2016). D-fructose being an intermediate in 5-HMF generation, the enhanced isomerization rate could explain the high amounts of 5-HMF observed in seawater. Since D-fructose is more readily converted into products than D-glucose, this improved isomerization could also explain the observed conversion.

### Impact of Salts Concentrations and Seawater on Furanic Compounds and Organic Acids Production

Monosaccharides dehydration results showed significant differences in molar conversion efficiency into furanic compounds and organic acids. These differences are explained by the effect of salts type and concentration. So analyses under different salts composition for each monosaccharide were completed by a PCA.

**Figure 8:**
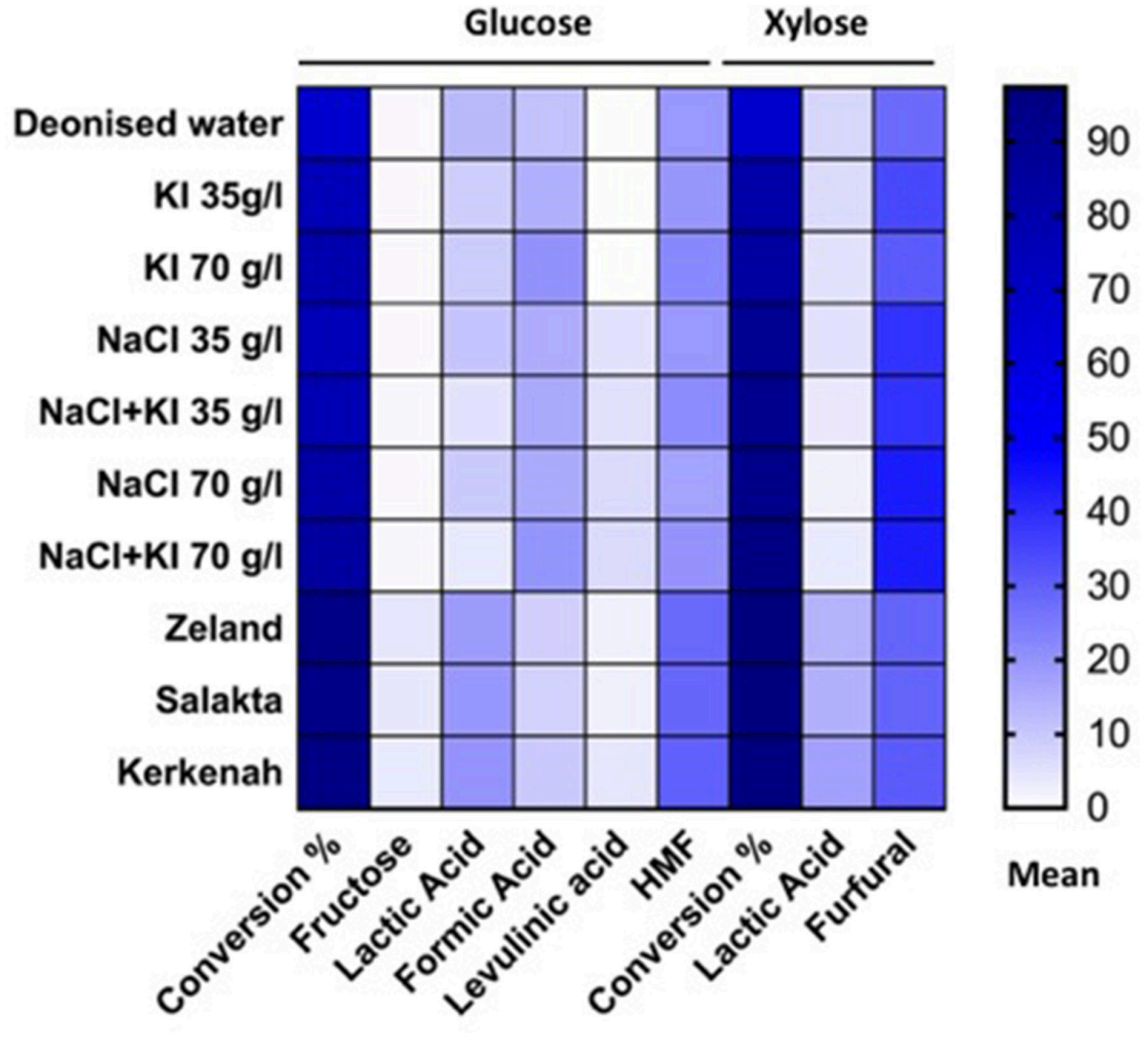
Principal component analysis of molar conversion of D-xylose and its molar conversion efficiency into furfural and lactic acid under different salts concentration and seawater treatment. **(A)** Correlation circle: red lines represent parameters (active variables) and blue lines show the treatments (supplemental variables), **(B)** Observations, and **(C)** correlation plot. *P < 0.05; ***P < 0.001.

Concerning D-xylose, PCA has been performed by considering three parameters and ten treatments. Figures 8A–C show the correlation circle, the observations and the correlation plot. The correlation circle explained 95.20% of the total variation. The first component F1 (axis 1) explained 54.22% of the variation, followed by 40.99% for the second component (axis 2). Observations explained 97.87% of total variation. PCA1 and PCA2 explained 56.684 and 41.186%, respectively. The correloplot is a correlation matrix which informs about correlation intensity. Conversion was positively correlated with furfural production (Pearson correlation coefficient *R* = 0.32) and with lactic acid appearance (*R* = 0.33), which could be explained by the pH shift during the treatment. Conversion of D-xylose to organic acids progressively decreases pH, favoring the generation of products by acid catalyzed pathways like the production of furfural. Lactic acid was positively correlated with seawater media with a correlation coefficient of 0.4 for Zealand and Salakta seawater and of 0.7 for Kerkenah seawater. This perfectly matches previous analysis. Seawater is a catalytic media to produce lactic acid from monosaccharides. 2-furfural showed a positive correlation with NaCl compared to KI. It presents a better correlation with the highest concentration of NaCl.

**Figure 9:**
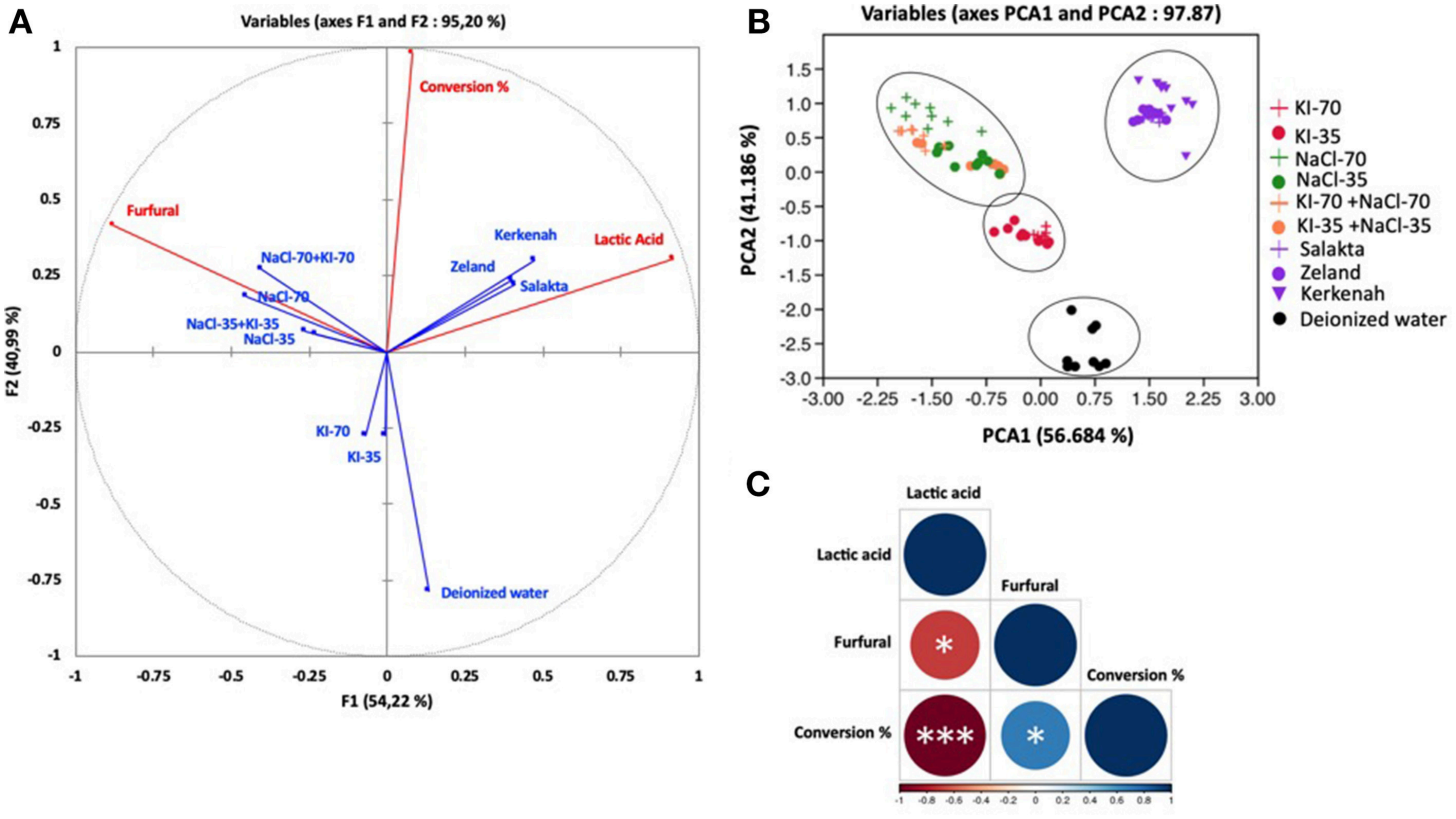
Principal component analysis of molar conversion of D-glucose and its molar conversion efficiency into fructose, formic acid, lactic acid, levulinic acid, and HMF under different salts concentration and seawater treatment. **(A)** Correlation circle: red lines represent parameters and blue lines refer to the treatments, **(B)** Observations, and **(C)** correlation plot. *P < 0.05; **P < 0.01; ***P < 0.001.

Concerning D-glucose, results are depicted in Figures 9A–C. Observations explained 89.848% of the total variation (PCA1 69.04%, PCA2 20.79%) followed by the correlation circle (F1 66.16% and F2 20.69%). PCA effected by considering six parameters and ten treatments. Conversion was highly positively correlated with HMF production and fructose formation with a Pearson correlation coefficient of 0.82 and 0.87, respectively. It was also moderately correlated with lactic acid (*R* = 0.61) and weakly correlated with levulinic acid (R = 0.24). On the other hand, negatively correlated with formic acid (*R* = −0.48). So, PCA analyses confirm clearly that HMF is positively correlated to fructose, which explains the increase of HMF production when fructose yield enhanced in seawater media. Fructose and HMF are highly negatively correlated to formic acid (*R* = 0.86 and *R* = 0.795, respectively) and weakly to levulinic acid production (*R* = −0.068 and *R* = −0.148, respectively). This negative correlation evinces HMF rehydration into formic acid and levulinic acid as shown in Figure 3, and thus HMF yield decreases when formic acid and levulinic acid were formed. Possibly levulinic acid reacts in the media and transformed into another molecule as its yield is lower than that of formic acid. Lactic acid is highly negatively correlated to formic acid (*R* = −0.895), and this may be explained by the different reactions types to form them from glucose based on Figure 3. Unlike lactic acid and furfural production from D-xylose, lactic acid generation and 5-HMF appearance from D-glucose are positively correlated. This observation does not seem consistent with their formation pathways requiring different pH conditions. D-glucose is however highly resilient to conversion in a slightly acidic medium. 5-HMF production is therefore facilitated by neutral or slightly alkaline conditions which favor D-glucose transformation to D-fructose by the Lobry Debruin VanEkenstein mechanism prior to the dehydration reaction.

## Conclusions

**Figure 10:**
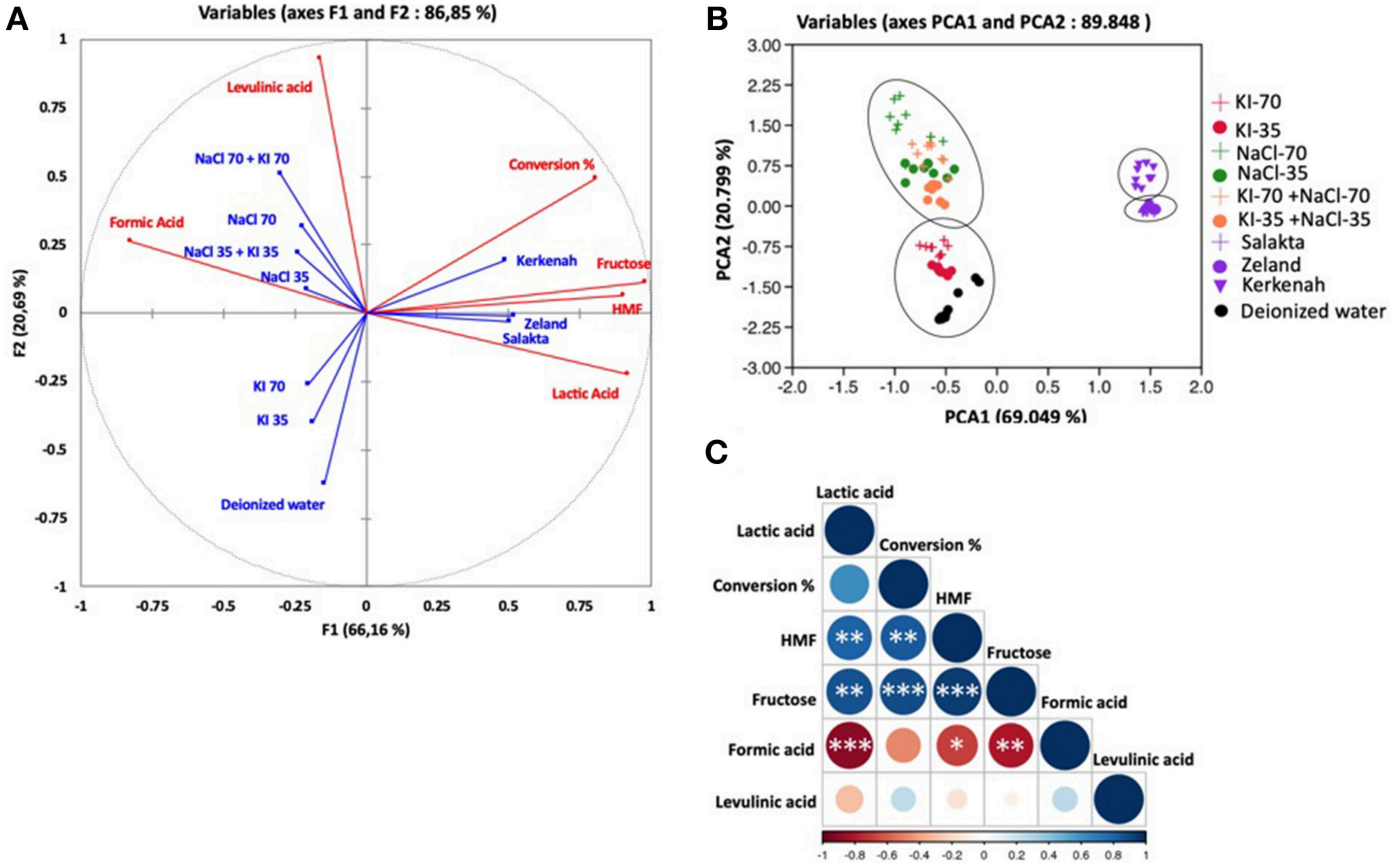
Seawater is a cheap, abundant and safe medium able to improve conversion and dehydration reactions of monosaccharides compared to salts aqueous medium.

This study was focused on the different products generated during hydrothermal treatment of D-xylose and D-glucose at 211°C in deionized water, NaCl and KI solutions at various concentrations and seawater. Typical reaction products were mixtures of lactic acid, formic acid, levulinic acid, 5-HMF and 2-F. Figure 10 shows the main products of each sugar under different treatments.

2-F yield from D-xylose is especially enhanced by the addition of NaCl and increases with salt concentration. The addition of NaCl to a D-glucose solution improves 5-HMF rehydration to levulinic and formic acid. KI favored other pathways toward formic acid production from D-glucose. 5-HMF production was improved in seawater as well as D-fructose concentration which indicates that this medium can promote isomerization. Seawater is consequently a cheap and suitable medium to perform C5- and C6-sugars conversion to furanic and acidic products especially to lactic acid production. The use of seawater in combination with dehydration catalysts or in biphasic systems could offer interesting performances while sparing drinkable water resources and should be investigated for developed costal countries.

## Author Contributions

All experiments, data analysis, discussion, and the manuscript preparation were done by MK. AR carried out the development and discussion of this work. TI assisted with discussion. HA conducted statistical analyses and figures preparations. NR and TB realized the manuscript recension. All authors have approved the submitted manuscript.

### Conflict of Interest Statement

The authors declare that the research was conducted in the absence of any commercial or financial relationships that could be construed as a potential conflict of interest.
