# Supplementary material for: Hydrothermal Dehydration of Monosaccharides Promoted by Seawater: Fundamentals on the Catalytic Role of Inorganic Salts
Source: Front Chem. 2019 Mar 22;7:132. doi: 10.3389/fchem.2019.00132 (PMC6440317; doi:10.3389/fchem.2019.00132)
Supplement: Supplementary file 1 [file Data_Sheet_2.pdf]

Supplementary Material

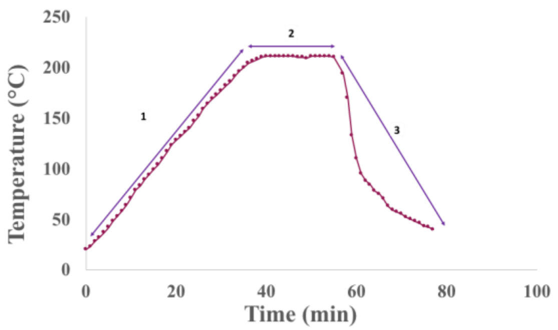

Figure s1: Temperature evolution curve of monosaccharide dehydration in hydrothermal condition in a Series 5500 Parr reactor. 1: The ramp time, 2: treatment at 15min and 3: cooling time.

$$\text{Monosaccharide conversion (\%)} = \left(1 - \frac{\text{Residual moles of monosaccharide}}{\text{Starting moles of monosaccharide}}\right) \times 100\ \%$$

$$\text{Lactic acid molar conversion efficiency from monosaccharide (\%)} = \left(\frac{\text{moles of lactic acid produced}}{\text{moles of starting monosaccharide}}\right) \times 100\ \%$$

$$\text{2-Furfural molar conversion efficiency from D-xylose (\%)} = \left(\frac{\text{moles of 2-Furfural produced}}{\text{moles of starting D-xylose}}\right) \times 100\ \%$$

$$\text{HMF molar conversion efficiency from D-glucose (\%)} = \left(\frac{\text{moles of HMF produced}}{\text{moles of starting D-glucose}}\right) \times 100\ \%$$

$$\text{Formic acid molar conversion efficiency from D-glucose (\%)} = \left(\frac{\text{moles of formic acid produced}}{\text{moles of starting D-glucose}}\right) \times 100\ \%$$

$$\text{Levulinic acid molar conversion efficiency from D-glucose (\%)} = \left(\frac{\text{moles of levulinic produced}}{\text{moles of starting D-glucose}}\right) \times 100\ \%$$

$$\text{Fructose molar conversion efficiency from D-glucose (\%)} = \left(\frac{\text{moles of fructose produced}}{\text{moles of starting D-glucose}}\right) \times 100\ \%$$

Figure s2: The yields of converted products derived from monosaccharides

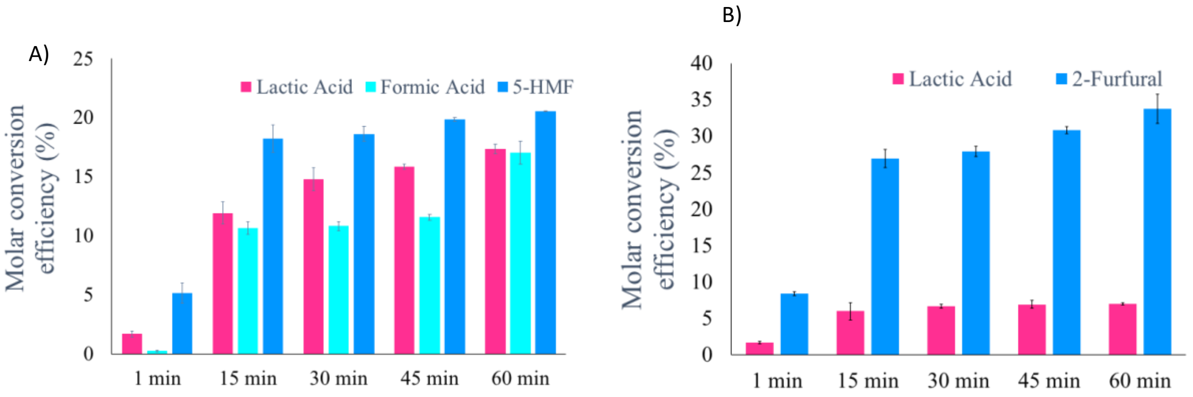

Figure s3: The resulting products of monosaccharide decomposition in deionized water at 211°C for different times. A) Conversion of D-glucose to Lactic acid, formic acid and 5-HMF. B) Conversion of D-xylose to Lactic acid and 2-Furfural. Figures are the means of three replicates, error bars: Standard deviation (SD).

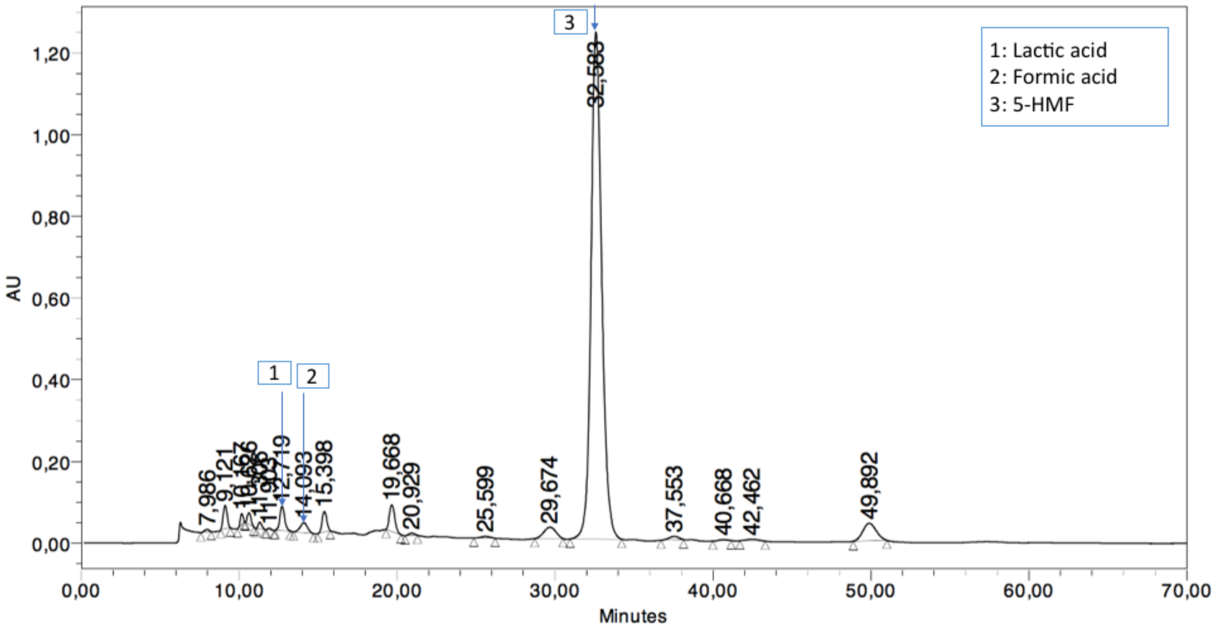

Figure s4: Chromatogram HPLC-UV (210nm) for D-glucose dehydration in deionized water at 211°C. Samples are diluted X 10.

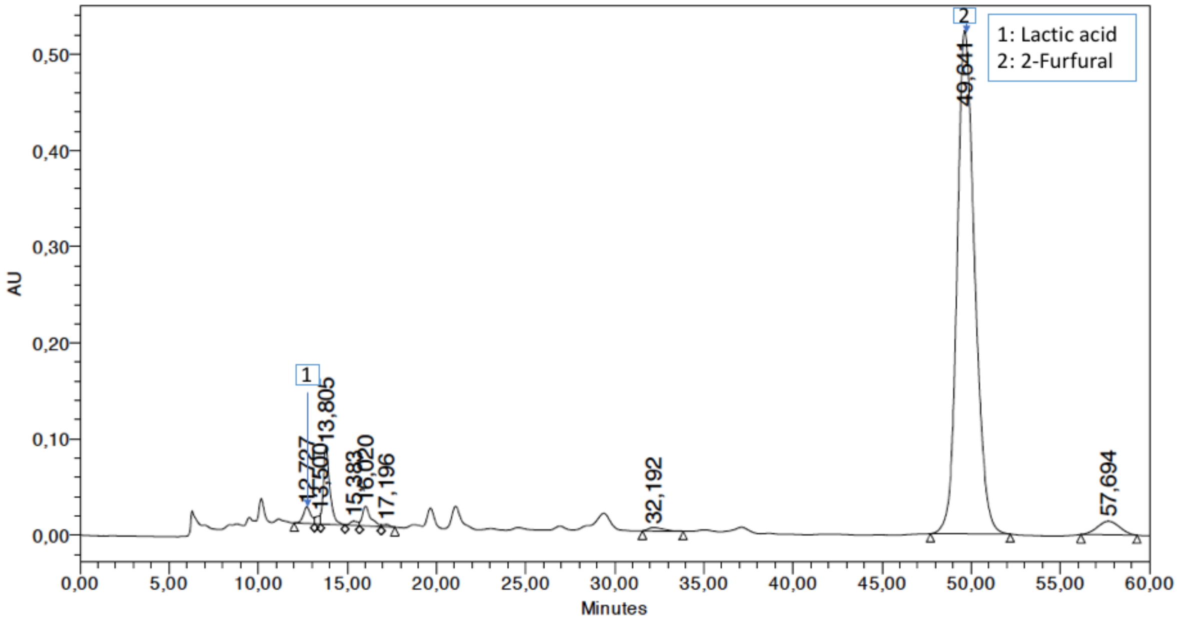

Figure s5: Chromatogram HPLC-UV (210nm) for D-xylose dehydration in deionized water at 211°C. Samples are diluted X 20.

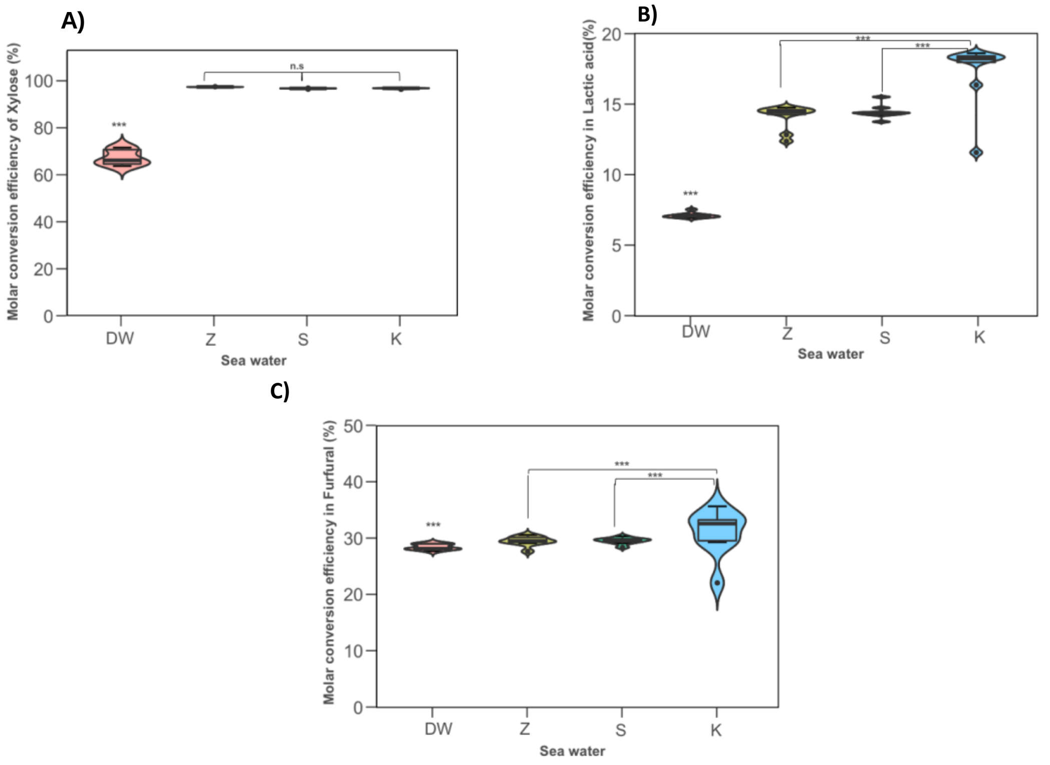

Figure s6: The effect of seawater on D-xylose dehydration: A) molar conversion efficiency of D-xylose, B) molar conversion efficiency to Lactic acid and C) molar conversion efficiency to 2-Furfural. DW: deionized water, Z: Zeland, S: Salakta, K: Kerkenah. *P<0.05; ***P<0,001.

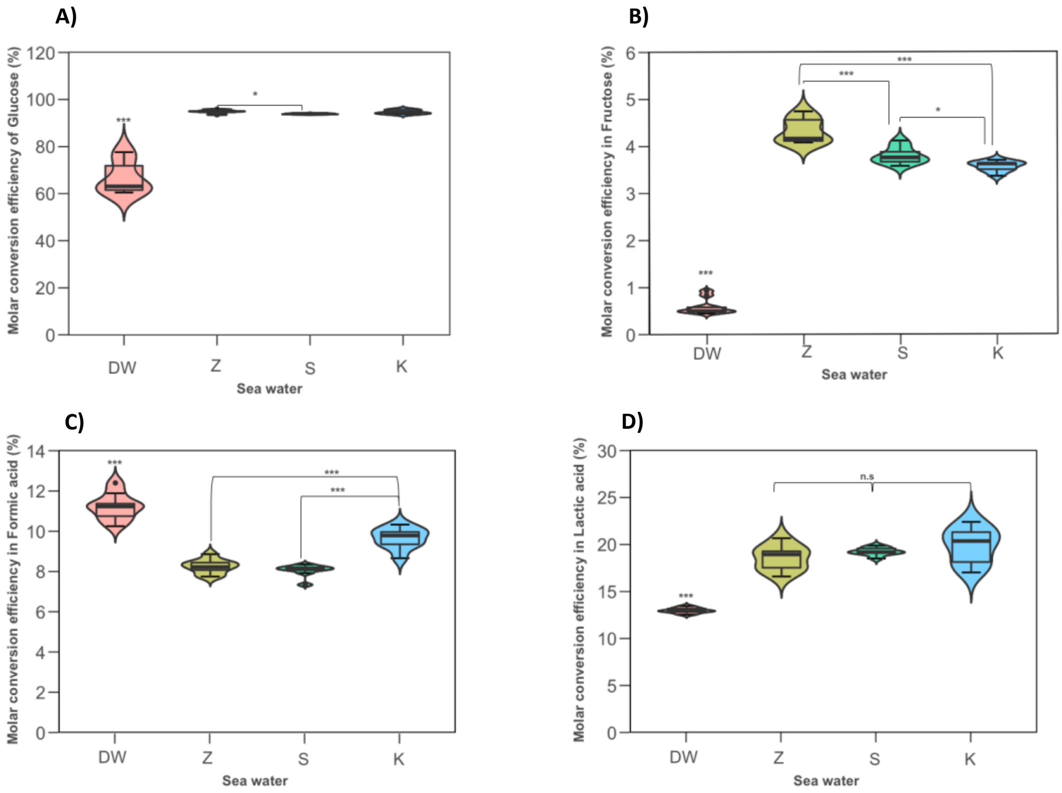

E) F)

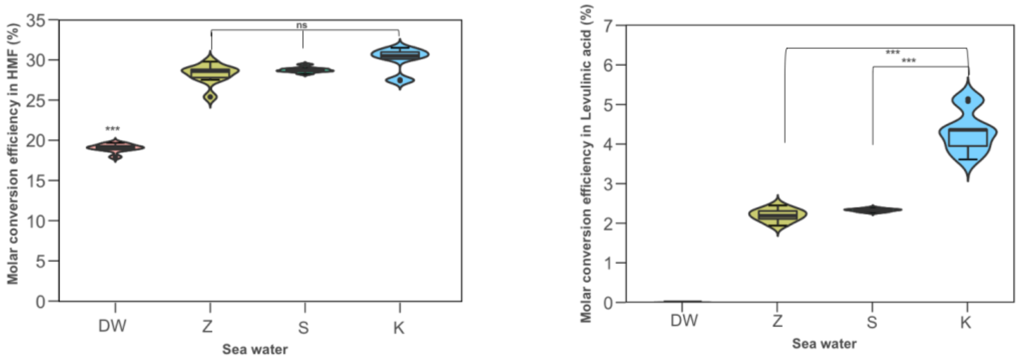

Figure s7: The effect of seawater on D-glucose dehydration: A) molar conversion efficiency of D-glucose, B) molar conversion efficiency to fructose, C) molar conversion efficiency to formic acid, D) molar conversion efficiency to lactic acid, E) molar conversion efficiency to HMF and F) molar conversion efficiency to levulinic acid. DW: deionized water, Z: Zeland, S: Salakta, K: Kerkenah. *P<0.05; ***P<0,001..
